# Proteomic profiling and functional characterization of early and late shoulder osteoarthritis

**DOI:** 10.1186/ar4369

**Published:** 2013-11-06

**Authors:** John Paul Wanner, Roopashree Subbaiah, Yelenna Skomorovska-Prokvolit, Yousef Shishani, Eric Boilard, Sujatha Mohan, Robert Gillespie, Masaru Miyagi, Reuben Gobezie

**Affiliations:** 1 Department of Orthopaedics, Case Western Reserve University, School of Medicine, Cleveland, OH, USA; 2 Centre de Recherche en Rhumatologie et Immunologie, Centre de Recherche du Centre Hospitalier Universitaire de Québec, Faculté de Médecine de l’Université Laval, Québec, QC G1V 4G2, Canada; 3 Laboratory for Integrated Bioinformatics, RIKEN Center for Integrative Medical Sciences (IMS-RCAI), RIKEN Yokohama Institute, Kanagawa, 230 0045, Japan; 4 Case Center for Proteomics and Bioinformatics, Case Western Reserve University, School of Medicine, Cleveland, OH, USA; 5 Cleveland Shoulder Institute, University Hospitals of Cleveland, 5885 Landerbrook Drive, Monarch Center, Mayfield Heights, OH, 44121, USA

## Abstract

**Introduction:**

The development of effective treatments for osteoarthritis (OA) has been hampered by a poor understanding of OA at the cellular and molecular levels. Emerging as a disease of the 'whole joint’, the importance of the biochemical contribution of various tissues, including synovium, bone and articular cartilage, has become increasingly significant. Bathing the entire joint structure, the proteomic analysis of synovial fluid (SF) from osteoarthritic shoulders offers a valuable 'snapshot’ of the biologic environment throughout disease progression. The purpose of this study was to identify differentially expressed proteins in early and late shoulder osteoarthritic SF in comparison to healthy SF.

**Methods:**

A quantitative ^18^O labeling proteomic approach was employed to identify the dysregulated SF proteins in early (n = 5) and late (n = 4) OA patients compared to control individuals (n = 5). In addition, ELISA was used to quantify six pro-inflammatory and two anti-inflammatory cytokines.

**Results:**

Key results include a greater relative abundance of proteins related to the complement system and the extracellular matrix in SF from both early and late OA. Pathway analyses suggests dysregulation of the acute phase response, liver x receptor/retinoid x receptor (LXR/RXR), complement system and coagulation pathways in both early and late OA. The network related to lipid metabolism was down-regulated in both early and late OA. Inflammatory cytokines including interleukin (IL) 6, IL 8 and IL 18 were up-regulated in early and late OA.

**Conclusions:**

The results suggest a dysregulation of wound repair pathways in shoulder OA contributing to the presence of a 'chronic wound’ that progresses irreversibly from early to later stages of OA. Protease inhibitors were downregulated in late OA suggesting uncontrolled proteolytic activity occurring in late OA. These results contribute to the theory that protease inhibitors represent promising therapeutic agents which could limit proteolytic activity that ultimately leads to cartilage destruction.

## Introduction

Arthritis is a disabling and debilitating disease that is estimated to affect 50 million people or 22% of adults in the United States, with 21 million or 9% of all adults having some limitation due to the disease [1]. As the population advances in both size and age, it has been projected that 67 million adults, aged 18 years or older, will be affected by this disease by 2030 [2]. With current annual costs of $128 billion, these statistics demonstrate the ever-increasing burden osteoarthritis (OA) will have on the healthcare industry [1]. Given the alarming numbers and increasing economic strain, there is an urgent need to identify key biomarkers that allow for targeted drug therapeutics.

A great deal of variance exists in regards to the prevalence of OA in the body’s joints. It has been found that the incidence of OA is disproportionately concentrated in weight-bearing joints such as the hip and knee. Hip and knee replacement surgeries accounted for 35% of total arthritis-related procedures during hospitalization in the last year [3]. In 2004, there were 454,652 total knee replacements, 232,857 total hip replacements, 41,934 total shoulder replacements and 12,055 other joint replacements in the United States [4]. While the clinical outcome of OA is clearly similar in weight-bearing and non-weight-bearing joints, the biologic similarity is less recognized.

Many candidate molecules have been identified as potential promoters and regulators of OA progression, but none have proven to be both necessary and sufficient. Various matrix metalloproteinases (MMPs) have been implemented as key contributors to the erosion of articular cartilage including MMP-3 [5]. Several proinflammatory cytokines have been implicated as possible therapeutic targets with existing research indicating that IL-1β, IL-6 and TNF-α are the major proinflammatory cytokines in OA [6]. Synovial macrophages and macrophage released cytokines are known to cause destruction of articular cartilage [7]. The complement system has also been described by Wang *et al.* as being a key contributor to OA progression. Their group found that the dysregulation of complement in mice (component 5 (C5), C6 or the complement regulatory protein (CD59a) proved to be key factors in the onset and pathogenesis of OA [8].

Although the clinical manifestations - cartilage degeneration, pathological changes in the subchondral bone and irritation of the synovium - are well-documented, it is evident that OA remains poorly understood at the cellular and molecular level. Various etiologic factors including age, sex, genetics, trauma, overuse, anatomical abnormalities and obesity have been identified and their interplay culminates in patient reports of pain and dysfunction [9]. Traditionally, OA was thought to be a disease concentrated in the articular cartilage but, as described above, the complexity of OA is now well appreciated and it is currently viewed as a disease of the whole joint. Bathing all structures within the joint, synovial fluid (SF) provides a snapshot of the entire biologic environment throughout disease progression. With many tissues contributing to OA pathogenesis, it is crucial that the entire environment be studied throughout disease progression so that the contribution of each tissue might be appreciated.

Mass spectrometry allows for the rapid detection and quantification of proteins composing specific tissues within the body. This technique has been refined and developed to a point where increases in sensitivity and resolution have resulted in higher accuracy when identifying peptides in complex tissues. This study aims to analyze early and late stages of OA progression through the proteomic analysis of SF and then compare these biologic environments to that of a normal shoulder control group. Studying this snapshot of the biologic environment throughout disease progression should help to identify key pathogenic contributors.

## Methods

### Patient cohort

Informed consent was obtained from 90 patients, from whom 59 samples were collected. The patient population studied consisted of 16 individuals (8 male and 8 female), with an average age of 58 years (range 37 to 74). Samples were collected prior to trocar placement or initial incision in order to minimize blood contamination of the accessed joint space. All samples for the study were collected from patients within our tertiary care referral center. All aspects of sample collection were approved by the University Hospitals Institutional Review Board in Cleveland, Ohio.

Control individuals: the control group consisted of six patients (three male/three female) with an average age of 54 years (30 to 60). These patients were without any prior history of shoulder surgery, blood dyscrasias, cancer, chondrocalcinosis or corticosteroid injection within the past eight weeks. Radiographic and/or magnetic resonance (MR) imaging demonstrated a normal joint space without any visible degenerative changes. Samples that were free from visible blood contamination and consisted of a minimum of 300 μl were collected.

Patients with early OA: the early-stage OA group consisted of five patients (two male/three female) with an average age of 57 years (50 to 66). Patients who displayed early pathologic changes in synovium and articular cartilage were identified through radiographic, MR or arthroscopic imaging. Patients undergoing arthroscopic labral repair were preferentially used for this group as this tissue is relatively avascular and thus, least likely to contribute a confounding inflammatory response to our proteomic profile. Again these patients were without clinically significant shoulder trauma, blood dyscrasias, cancer, chondrocalcinosis or corticosteroid injection within the past eight weeks. Samples that were free from visible blood contamination and consisted of a minimum of 300 μl were collected.

Patients with late OA: the late stage OA group consisted of five patients (two male/three female) with an average age of 64 years (47 to 74) who displayed advanced degenerative changes in all aspects of the glenohumeral joint such that a total shoulder replacement was necessary. Exclusion criteria based on the above co-morbidities were also used for this cohort. Samples that were free from visible blood contamination and consisted of a minimum of 300 μl were collected.

### Materials

We purchased oxygen-18 (^18^O)-water (97%) from Isotec (Miamisburg, OH, USA). HPLC solvents were purchased from Fisher scientific Honeywell, B&J HPLC Water Muskegon, MI, USA. The ^16^O-water used in all experiments was HPLC grade. The multiple affinity removal column was purchased from Agilent Technologies (Palo Alto, CA, USA). Perfluorooctanoic acid (PFOA) was purchased from TCI America (Portland, OR, USA). All other reagents were of the highest quality commercially available.

### SF sample preparation

An 18-gauge needle was inserted into the glenohumeral joint and up to 4 mL of SF was removed. A stock solution consisting of one tablet of complete protease inhibitor cocktail tablets was mixed in 2 mL of 1 × PBS (Roche Applied Science, Indianapolis, IN, USA). This solution was then added to the collected SF sample in a 1:4 dilution in a 5-mL cryogenic tube. The sample was vortexed and then centrifuged at 402 × *g* for 10 minutes to remove any blood contamination. The SF was then removed with a pipette and transferred to a separate cryogenic tube and snap-frozen in liquid nitrogen and placed in an -80°C freezer.

At the time of processing, the samples were thawed on ice and the protein amount was estimated with a DC protein assay kit (Bio-Rad, Hercules, CA, USA) using bovine serum albumin as a standard protein. To this, 1 μg/mL of bovine hyaluronidase (Sigma-Aldrich, St. Louis, MO, USA) was added and the sample was placed in a 37°C water bath for 3 hours.

### Immunodepletion

Each sample was then processed to deplete the 14 most abundant proteins in SF using an Agilent high capacity multiple affinity removal spin cartridge according to manufacturer’s instructions. Buffer exchange to 0.1 M ammonium bicarbonate (ABC) was performed using centrifugal filter units -3 K (Amicon^®^ Ultra, Millipore, Carrigtwohill, Co.Cork, Ireland) and centrifuged at 6,000 × *g* for 20 minutes at 4°C to bring the pH to an alkaline level. This procedure was repeated three times for each sample. SDS-PAGE was performed to ensure that the depletion column was working properly throughout the experiment.

### Proteomic sample preparation for immunodepleted SF

Immunodepleted samples (n = 5) from control/healthy groups were pooled and labeled with ^18^O and individual samples from experimental groups were labeled with ^16^O. The procedure used for one set of pooled control/individual diseased samples is as described earlier [10]. Protein amount was estimated with a DC protein assay kit (Bio-Rad) using bovine serum albumin as a standard protein. We reduced 100 μg of low abundant proteins from pooled healthy samples and the individual diseased sample and these were alkylated by using 21 mM triethyl phosphine (TEP) and 58 mM iodoethanol (IE). The samples were then dried in a Speed-vac concentrator and dissolved in 100 μL of 0.1 M ABC containing 1% PFOA. Trypsin was added in the ratio of 1 μg per 50 μg of protein. The samples were placed in a 37°C water bath for 18 hours. A 1:1:1 solution of ethanol, ethyl acetate and HPLC-grade water with 0.1% TFA was added to the peptide digest and dried in a Speed-vac concentrator to remove PFOA [10]. The digest was then subjected to ^18^O labeling as follows: 40 μL of ^18^O buffer (50 mM ammonium acetate, pH 5.5) and trypsin were added to the dried samples and the process was repeated for the ^16^O samples. The samples were incubated at 37°C for 18 hours. The reaction was stopped by drying the samples and adding 50 mM ABC containing isopropyl alcohol (final concentration = 70%). The enzyme was inactivated by reducing (TEP/acetonitrile) at 45°C for 1 hour and then alkylating (iodoethanol/acetonitrile) in the dark at 45°C for 2 hours as described previously [10]. The ^18^O- and ^16^O-labeled samples were mixed in a 1:1 ratio and chromatographed on a C18 column (3.5 μ, 4.6 × 150 mm, WatersXterra^®^ MS column Waters XTerra colum XTerra^®^, MS C18 3.5 um 4.6 × 150 mm Waters corporation 34 maple street Milford, Massachussets) using a linear gradient of acetonitrile from 0% to 90% in 30 mM ammonium formate (pH 10.0) over a period of 60 minutes at a flow rate of 400 μl/minute in a Thermo Finnigan HPLC system. The effluent was monitored by the absorbance at 215 nm. The effluent was collected every minute. The peptide fractions were dried in a Speed-vac, dissolved in 0.1% formic acid, pooled into 10 fractions, and stored until analyzed by liquid chromatography tandem mass spectrometry (LC-MS/MS). A total of five biological replicates from early and late sets were analyzed by mass spectrometry. Out of five biological replicates from late-stage OA samples, one had to be discarded due to heavy contamination by blood cells.

### Proteomic sample preparation for SF proteins bound to high capacity multiple affinity removal spin cartridge

SF proteins bound to the high capacity multiple affinity removal spin cartridge were also analyzed by fractionating proteins by SDS-PAGE followed by in-gel ^18^O labeling. Protein concentrations were measured using the Biorad DC protein assay kit. Equal concentrations of proteins from the control and diseased sample sets were separated on 4 to 20% polyacrylamide gel and visualized by colloidal Coomassie stain. Eight slices of exact equal size (excluding serum albumin protein band) from healthy and diseased sample sets were excised and subjected to trypsin digestion as described earlier [11]. In short, gel slices were subjected to reduction/alkylation using triethyl phosphine/iodoethanol and then de-stained. Gel pieces were saturated with trypsin buffer and digested with trypsin overnight. The peptides were extracted, subjected to labeling and mixed as described earlier in this manuscript. Control/healthy peptides were treated with ^16^O and diseased peptides were treated with ^18^O water, and mixed, and this was subjected to LC-MS/MS.

### LC-MS/MS analysis

The protein digests were analyzed by LC-MS/MS using an UltiMate 3000 LC system (Dionex, San Francisco, CA, USA) interfaced to an LTQ-Orbitrap XL mass spectrometer (Thermo-Finnigan, Bremen, Germany). The platform operated in the nano-LC mode using the standard nano-ESI API stack fitted with a picotip emitter (uncoated fitting, 10-μm spray orifice, New Objective, Inc., Woburn, MA, USA). The solvent flow rate through the column was maintained at 300 nL/minute using a 1:1,000 splitter system. The protein digests (5 μL) were injected into a reverse-phase C18 PepMap trapping column (0.3 × 5 mm, 5-μm particle size, Dionex Inc.), equilibrated with 0.1% formic acid/2% acetonitrile (v/v) and washed for 5 minutes with the equilibration solvent at a flow rate of 25 μL/minute using an isocratic loading pump operated through an auto-sampler. After the washing step, the trapping column was switched in line with a reversed-phase C18 Acclaim PepMap 100 column (0.075 × 150 mm, Dionex Inc.) and the peptides were chromatographed using a linear gradient of acetonitrile from 4.8% to 40% in aqueous 0.1% formic acid over a period of 90 minutes at the above-mentioned flow rate, such that the eluate was directly introduced to the mass spectrometer. An 80% acetonitrile elution step was subsequently performed for 5 minutes prior to resetting the analytical column to the initial equilibration conditions for 11 additional minutes at the end of the chromatographic run. The mass spectrometer was operated in a data-dependent MS to MS/MS switching mode, with the five most intense ions in each MS scan subjected to MS/MS analysis. The full scan was performed at 60,000 resolution in the Orbitrap detector and the MS/MS fragmentation scans were performed in the ion trap detector (IT) CID mode such that the total scan-cycle frequency was approximately 1 s. The threshold intensity for the MS/MS trigger was always set at 1,000 and the fragmentation was carried out using the CID mode and normalized collision energy (NCE) of 35. The data were collected in the profile mode for the full scan and centroid mode for the MS/MS scans. The dynamic exclusion function for previously selected precursor ions was enabled during the analysis such that the following parameters were applied: repeat count of two, repeat duration of 45 s, exclusion duration of 60 s and exclusion size list of 150. Xcalibur software (version 2.0.7, Thermo-Finnigan Inc.) was used for instrument control, data acquisition, and data processing.

### Mass spectrometry data analysis

Mass Spectrometry MS raw data files were searched against the Swiss-Prot (version 57) database using Mascot database search software (version 2.1.04, Matrix Science, London, UK) to identify peptides/proteins with the following parameters: S-hydroxyethylation of cysteine as a fixed modification, and oxidation of methionine to methionine sulfoxide and ^18^O labeling of peptide at the C-terminus as variable modifications. The mass tolerance for the precursor ion was set to 10 ppm, and for the product ion it was set to 0.8 Da. Strict trypsin specificity was applied, allowing for one missed cleavage. Peptides with a minimum score of 20 were considered as significant. In-house software employing a least-squares regression algorithm [12] was used for the calculation of ^16^O/^18^O peptide ratios. This software plots ^16^O/^18^O-peptide intensities of all peptides identified from the same protein, and the slope of the linear regression fit is used as a ^16^O/^18^O peptide ratio for that protein. Only proteins with an *r*^2^ ≥0.85 and a linear regression *F*-probability >0.85 are reported as quantified proteins. Proteins with *r*^2^ values or *F*-probabilities out of our range were manually investigated for possible peptide outliers. An obvious outlier was defined as a peptide whose removal changed the protein *r*^2^ value by more than 0.2 or increased the *F*-probability to >0.85. If an obvious outlier was detected, it was removed from the peptide list. Median normalization of the linear regression ratio was performed at the protein level within the sets to account for intra-experimental variation [13]. Following normalization, protein ratios from all runs were visualized using histograms. Scattered plot matrices of biological replicates were generated and the Pearson product moment correlation coefficient between biological replicates was calculated using Bioconductor [14]. Fold changes in the linear regression ratio of proteins were calculated using Excel. *P*-values were calculated using the limma package available in Bioconductor employing the empirical Bayes method [15]. The gplots package from Bioconductor was used to generate heat maps of protein sets [16]. The color coding is derived using the ranking of the log ratio in the distribution of measures for that sample. Shades of red represent downregulation, and shades of green represent upregulation. The intensity of the color is determined by the distance (in SD) from the mean of the trimmed distribution.

### Western blot analysis

Commercial antibodies used for western blot validation experiments were as follows: aggrecan (MA3-16888), tenascin (MA1-26779) and complement factor D (GAU 008-01-02) monoclonal antibodies (ThermoScientific Pierce Protein Research Products (Rockford, IL, USA).

SF (processed and unprocessed) was used for western analysis. SDS-PAGE was performed on 4 to 15% acrylamide gels with 30 μg protein applied per lane, and proteins were electrophoretically transferred to nitrocellulose membranes (Millipore, Billerica, MA, USA). Membranes were blocked with blocking buffer purchased from LI-COR (Lincoln, NE) and then incubated overnight at 4°C with primary antibody. The dilutions were performed as suggested by the manufacturer. The membranes were washed with PBS containing 0.1% Tween 20 and probed with secondary antibody (Antimouse 680 antibody from LICOR, diluted to 1:10,000) in blocking buffer containing 0.1% Tween 20 for 1 hour at room temperature. Proteins were detected using a LICOR Odyssey Scanner.

### Cytokine analysis by ELISA

SF was analyzed from five healthy subjects, and five with early and five with late stages of OA. SF cytokines were analyzed using commercially available kits. A kit containing IL-1β, IL-6, TNF-α, IL-8, IL-17, IL-10, IL-4 and IL-13 was procured from BioLegend (San Diego, CA, USA), and IL-18 was purchased separately from Medical & Biological laboratories (Nagoya, Japan). Assays were performed according to the manufacturer’s instructions. Manufacturer-reported precision and sensitivity data for these kits are as follows: IL-10 = <2 pg/mL, IL-13 = 5.8 pg/mL, IL-6 = 1.6 pg/mL, IL-4 = 0.6 pg/mL, TNF-α = 3.5 pg/mL, IL-1β = 0.5 pg/mL, IL-8 = 4.4 pg/mL, IL-17A = 11.5 pg/mL and IL-18 = 12.5 pg/mL.

### Bioinformatic analysis

Differentially expressed proteins were analyzed using IPA (version 8.8) (Ingenuity^®^ Systems, [17]) to determine the functions and pathways most strongly associated with the proteome set. Proteins with fold change values of 1.5 above and -1.5 below were uploaded into the application and analysis was performed to generate networks and canonical pathway analysis. The Benjamini-Hochberg multiple testing correction was applied to assess the significance of associations.

### Uric acid analysis

Uric acid concentration of SF samples was measured using the Uric Acid Assay Kit (No-KA1651, Abnova Corporation, Walnut, CA). The assay was performed according to the manufacturer instructions. The uric acid concentration of the samples was calculated as:

$$\begin{array}{ll} \left(\Delta A590\mathrm{nm}\;\text{sample}–\Delta A590\;\text{blank}\right)/ & \left(\Delta A590\mathrm{nm}\;\text{standard}–\Delta A590\;\text{blank}\right)\\ & \;\times 10\left(\mathrm{mg}/\mathrm{dL}\right). \end{array}$$

## Results

### Quantitative proteomic analysis of immunodepleted SF

**Figure 1:**
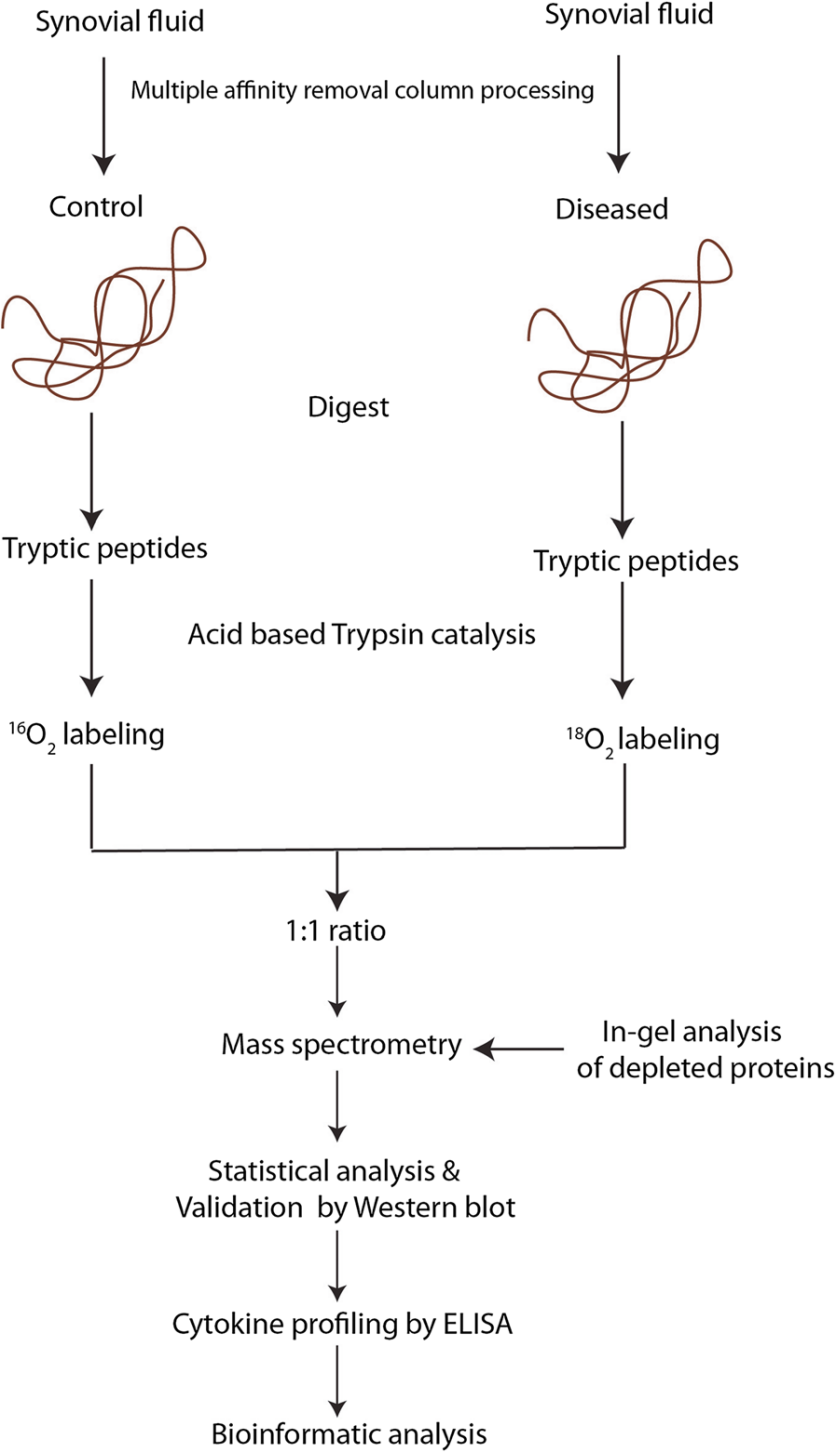
Schematic representation of proteomic analysis of shoulder joint synovial fluid.

To quantify differentially expressed proteins from osteoarthritic SF, we employed the proteolytic ^18^O labeling approach. A schematic representation of the SF proteomic workflow employed is depicted in Figure 1. SF is similar to serum with a small number of highly abundant proteins representing almost 95% of total protein content, thus making detection of low-abundance proteins difficult [18]. Consequently, we used a High Capacity Multiple Affinity Removal Spin Cartridge to remove 14 abundant proteins from SF. Healthy pooled samples (n = 6) were labeled with ^18^O and individual samples from early (n = 5) and late (n = 4) OA were labeled with ^16^O as described in the Methods section. The labeled peptide samples were first fractionated by high-pH reverse-phase chromatography, and then each fraction subjected to LC-MS/MS analysis. Proteins identified with one or more peptides with ≤1% false discovery rate (FDR) were used for subsequent statistical and bioinformatics analyses. The abundance ratios, presented as early or late OA patient over the healthy control group, and the molecular identities of these proteins in the five early OA versus control experiments and four late OA versus control experiments are summarized in Additional file 1: Table S1-Early1-5 and 1-Late1-4, respectively.

**Figure 2:**
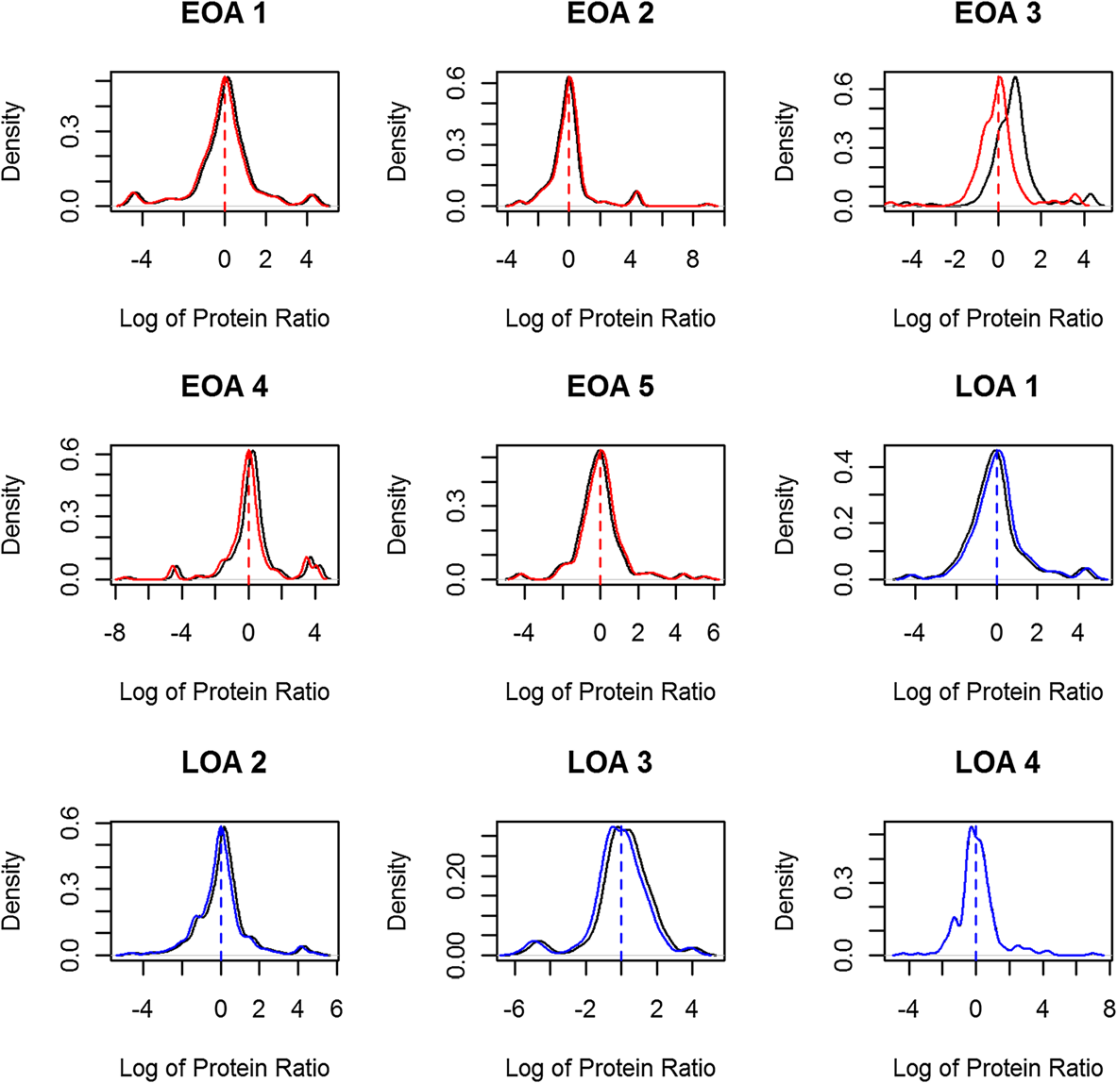
**Histogram plots of immunodepleted proteomic samples.** Sample sets early osteoarthritis (EOA) 1–5 are biological replicates from early-stage OA patients and sample sets late OA (LOA) 1–4 are biological replicates from late-stage OA patients. Log2 of protein ratios and normalized protein ratios are plotted on the x axis and estimated densities of log ratios on the y axis. The black curve represents experimental protein regression ratios; red and blue curves represent normalized ratios for early and late sample sets, respectively.

**Figure 3:**
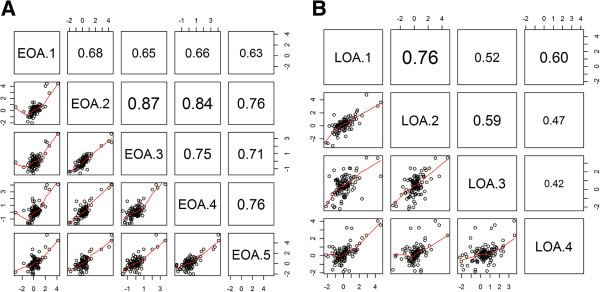
**Scatter plot matrix of immunodepleted proteomic samples.** Correlation scattered plot matrix analysis of early (EOA) **(A)** and late osteoarthritis (LOA) **(B)** sample sets. Pearson product moment correlation coefficient of each set with respect to other individual sets is represented. Clustering can be seen in several plots and positive correlation is seen between sample sets.

Additional file 1: Table S1 lists the complete data set including peptide sequences quantified, and Additional file 2: Table S2 (Early1-5 and Late1-4) lists only the UniProt accession number, protein name, number of peptides used in the protein quantification, abundance ratio, and fold changes (OA/healthy). The protein ratio and normalized ratios from each of the biological sets were visualized by histogram plots (Figure 2). We found 106 and 118 proteins in all the biological replicates within the early OA (Additional file 3: Table S3A) and late OA (Additional file 3: Table S3B) subgroups. To check the correlation between biological replicates, scatter plot matrices of these proteins in each biological set against other replicates within the subgroup were generated (Figure 3) and the Pearson product moment correlation was calculated. The correlation coefficient varied from 0.63 to 0.87 in early OA sets and 0.42 to 0.76 in late OA sets suggesting a positive correlation between the biological replicates within subgroups. Probability values for early and late OA sets are given in Additional file 3: Tables S3A and S3B, respectively.

### Quantitative proteomic analysis of SF proteins bound to high capacity multiple affinity removal spin cartridge

In addition to the immunodepleted samples, SF proteins bound to the high capacity multiple affinity removal spin cartridge were also analyzed to obtain the whole picture of proteins dysregulated in the diseased sample sets. Proteins were fractionated by SDS-PAGE and quantified by in-gel ^18^O labeling followed by LC-MS/MS. Of the 14 immunodepleted abundant proteins, Alpha-1-antitrypsin, Alpha-2-macroglobulin, serotransferrin and immunoglobulin molecules were differentially regulated in early OA sets (Additional file 4: Table S4A), and alpha-1-antitrypsin, apolipoprotein A-I, haptoglobin, alpha-1-acid glycoprotein 1, transthyretin and immunoglobulin molecules were differentially regulated in late OA sets (Additional file 4: Table S4B).

### Data analyses

**Figure 4:**
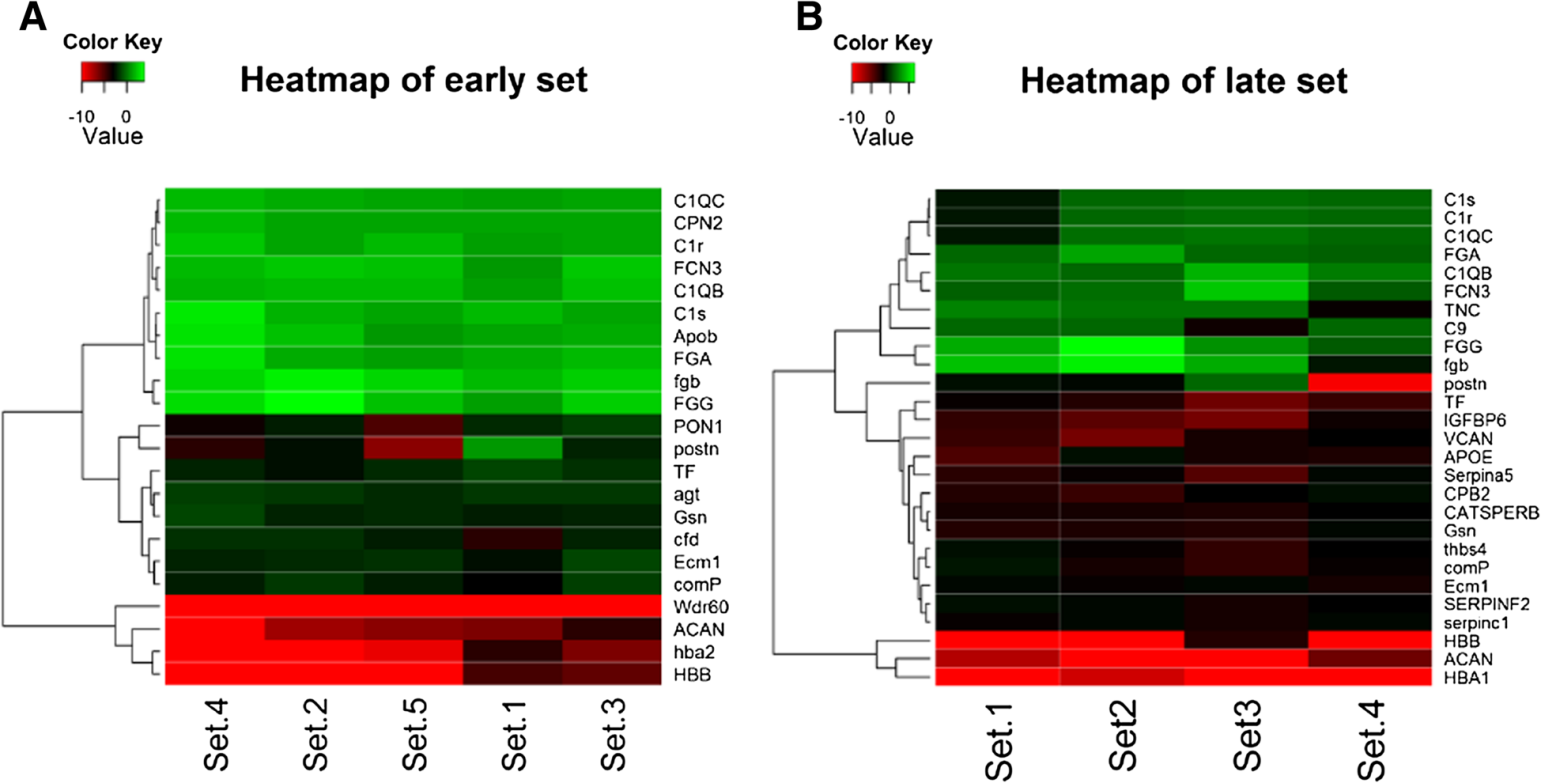
**Heat maps of early- and late-stage osteoarthritis (OA) sets.** Differentially expressed low abundant proteins from early OA (EOA) **(A)** and late OA (LOA) **(B)** sets are represented in the form of a heat map. The fold changes of proteins considered from biological replicates were above 1.5/below -1.5. Gene symbols are listed when known. Rows represent the proteins and columns represent the biological replicates in EOA and LOA. Upregulated and downregulated proteins are indicated in shades of green and red, respectively, where the intensity of the color is determined by the distance (in SD) from the mean of the trimmed distribution.

A total of 106 and 118 proteins were quantified across early- and late-stage OA samples, respectively (Additional file 2: Table S2), of which 31 and 38 proteins were differentially expressed with average fold-change values of ≥1.5 and ≤ -1.5 (Additional file 4: Table S4). These results suggest that most of the dysregulated proteins are already in place early in the development of the disease, characterizing a diseased proteome that is distinct from a healthy proteome. The heat map of proteomic alterations from the immunodepleted early and late OA sets show consistency across biological replicates (Figure 4). Analyses of altered proteins from immunodepleted samples as well as proteins bound to the high capacity multiple affinity removal spin cartridge (Additional file 5: Table S5) by The Database for Annotation, Visualization and Integrated Discovery (DAVID) showed complement proteins, proteoglycans and immunoglobulins to be dysregulated in both early and late OA sets, whereas serine protease inhibitors (serpins) were dysregulated only in late OA sets.

**Figure 5:**
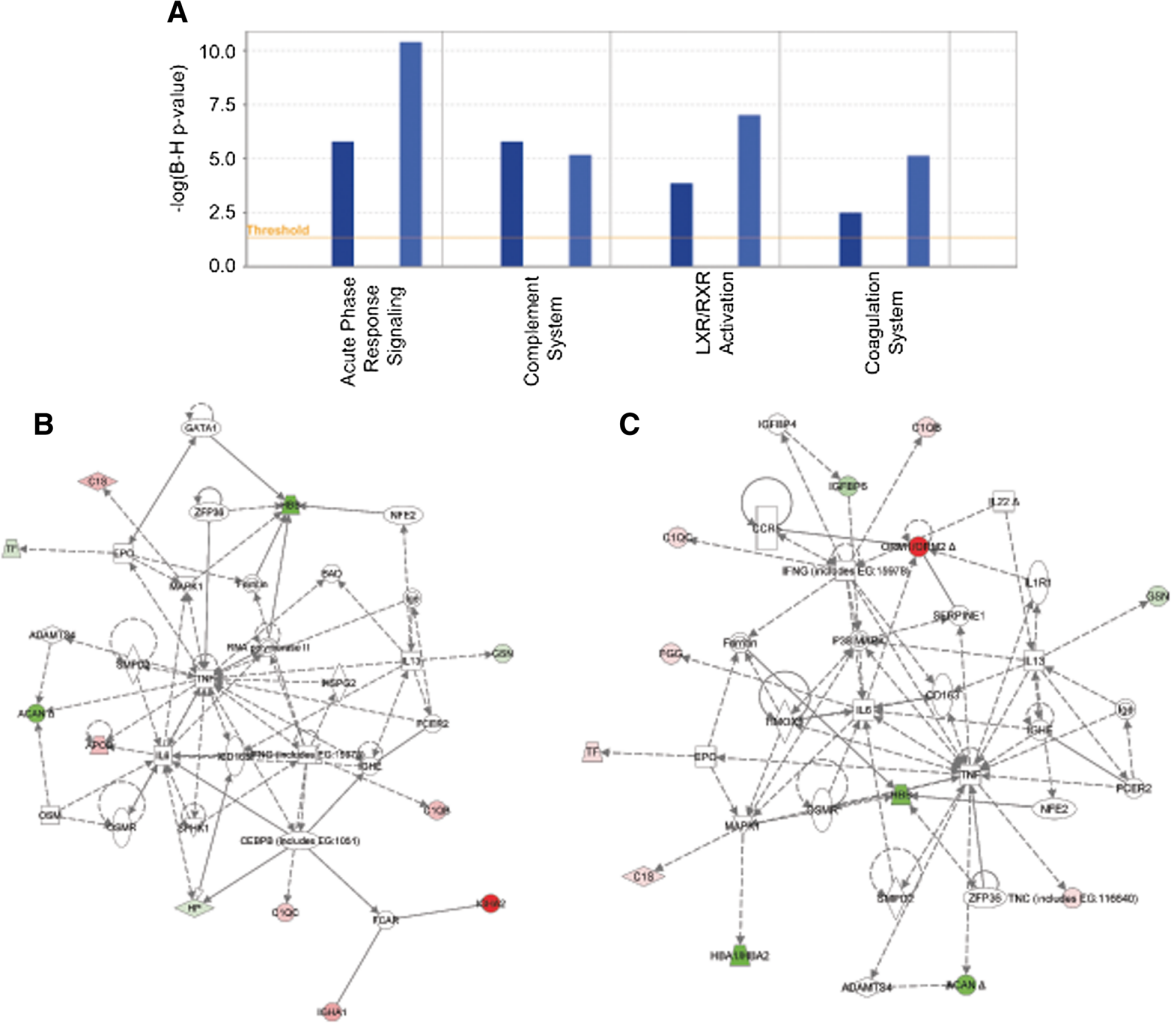
**Functional analysis of early and late sets. (A)** Comparison of pathway analysis between early and late sets. A probability value of 0.05 was applied for the analysis. **(B)** Top network *Cellular Development, Hematological System Development and Function, Hematopoiesis* associated with the early sample set **(C)** Top network *Organismal Injury and Abnormalities, Respiratory Disease, Cell-To-Cell signaling and Interaction* associated with the late sample set. Red color indicates higher level of expression in disease and green color indicates lower level of expression in disease. The names of the genes are in nodes. The shape of the nodes is related to gene function/family membership. The lines between the nodes represent the connectivity between genes. Solid lines indicate direct protein-protein interactions. Dotted lines represent indirect interactions.

Pathway analysis by Ingenuity systems suggests the dysregulation of wound repair pathways in SF in both the early and late OA subgroups (Figure 5A). The normal response to tissue injury occurs in three distinct overlapping stages. The first stage of wound repair is inflammation followed by activation of the complement system and the coagulation cascade [19]. The second stage is new tissue formation followed by remodeling of this new tissue. The top canonical pathway identified in both early and late OA sets was the acute phase response pathway, which is a rapid inflammatory response pathway seen in cases of tissue injury. Dysregulation of another inflammatory pathway, liver x receptor/retinoid x receptor (LXR/RXR) was seen in both early and late OA sets. Increased expression of complement molecules were also observed in both early and late OA sets. Activation of the coagulation pathway suggests an ongoing effort by the body to seal the wound formed in the shoulder joint. A network related to the hematological system was dysregulated in early OA, whereas organismal injury was dysregulated in late OA sets (Figure 5B and C). Networks related to lipid metabolism, molecular transport, small molecule biochemistry were downregulated in both early and late OA sets (data not shown). Pathways affecting new tissue formation/remodeling were not seen in either early or late OA sets.

### Cytokine analyses

**Figure 6:**
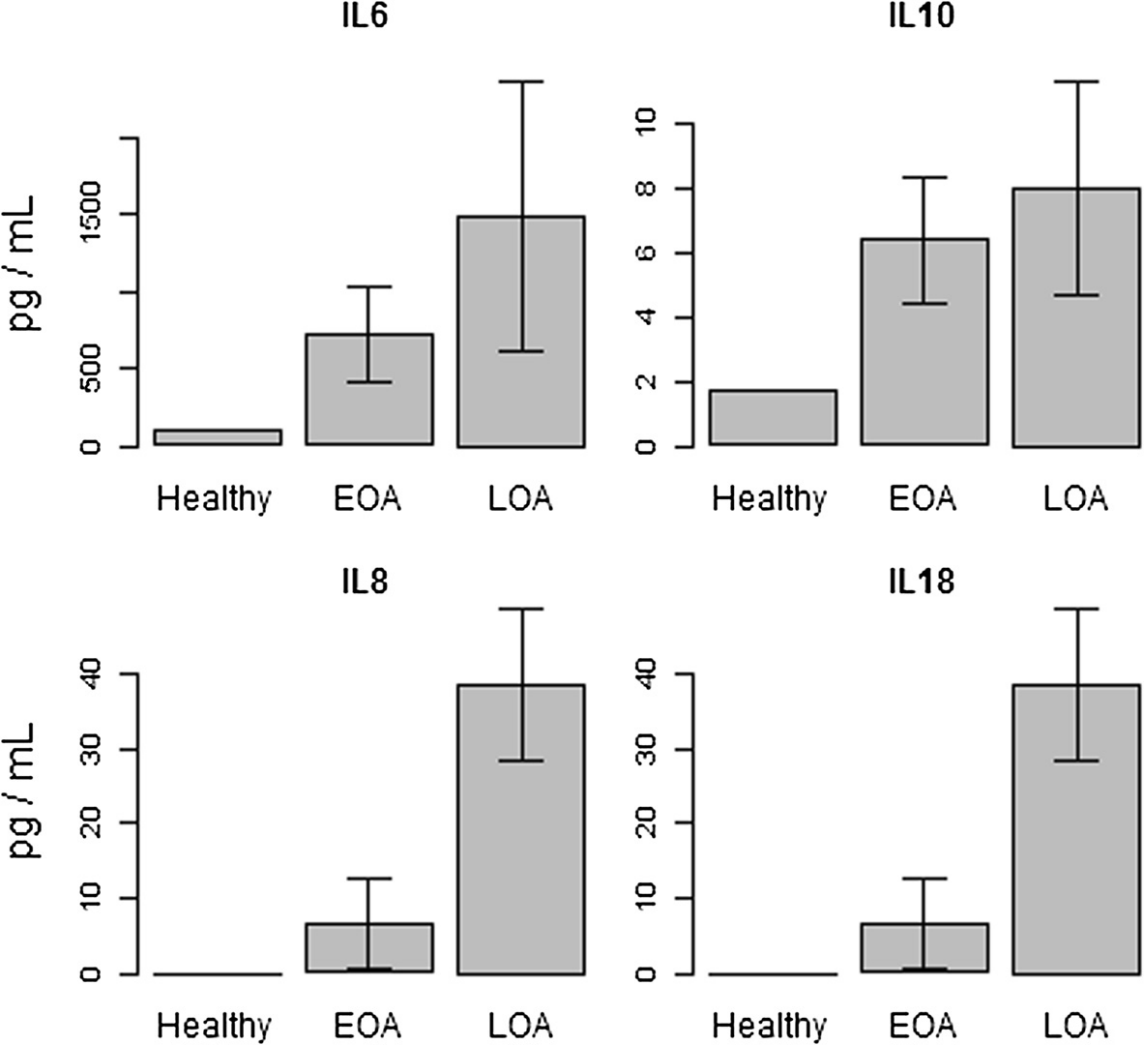
**Cytokine analyses.** Mean concentration values (n = 5) of cytokines are plotted with error bars representing SD. Values for healthy samples are from the pooled set (n = 6). EOA, early osteoarthritis; LOA, late osteoarthritis.

Proinflammatory cytokines play a major role in the pathogenesis of OA. SF from healthy, and early- and late-stage OA patients was quantified for six pro-inflammatory and two anti-inflammatory cytokines (Table 1). Mean cytokine concentrations of pro-inflammatory cytokines IL6, IL8 and IL18 were higher in diseased conditions when compared to healthy subjects (Figure 6). A modest increase was observed with anti-inflammatory cytokine IL10.

**Table 1:** Cytokine concentrations in synovial fluid from healthy subjects (n = 5, pooled), and patients with early- (n = 5) and late-stage (n = 5) osteoarthritis

**Pro-inflammatory interleukins**	**Sample (n = 5)**	**Mean (pg/mL)**	**SD**
**IL-6**	Healthy^a^	98.35	
	EOA	717.9	306.50
	LOA	1492.01	877.62
**IL-1β**	Healthy^a^	0	
	EOA	1.45	1.56
	LOA	4.64	3.29
**IL-8**	Healthy^a^	0	
	EOA	6.58	6.03
	LOA	38.63	10.35
**IL-17A**	Healthy^a^	0	
	EOA	0.02	0.05
	LOA	0	0
**TNF-alpha**	Healthy^a^	0	
	EOA	0.11	0.11
	LOA	0.51	0.63
**IL-18**	Healthy^a^	0.48	
	EOA	20.38	8.70
	LOA	37.29	15.36
**Anti-inflammatory interleukins**			
**IL-10**	Healthy^a^	1.68	
	EOA	6.39	1.95
	LOA	7.97	3.35

The numeric values depicted for EOA and LOA samples are mean ± SD (concentration range). ^a^Cytokine concentrations depicted for healthy synovial fluid samples (n = 5) are values obtained from pooled samples. EOA, early osteoarthritis; LOA, late osteoarthritis.

### Western blot validation

**Figure 7:**
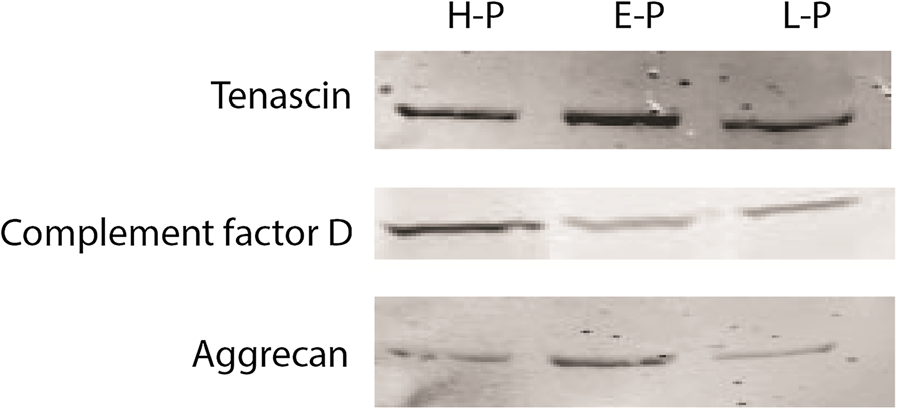
**Western blot analysis.** Tenascin, complement factor D and aggrecan were analyzed from pooled healthy (H-P), pooled early (E-P) and pooled late (L-P) sample sets: 50 μg of protein extract was loaded into each lane.

For verification of our mass spectrometry results, western blot analysis of three differentially expressed proteins (tenascin, complement factor D and aggrecan) was carried out (Figure 7). These proteins were selected on the basis of interesting biological function and high fold-change, as well as availability of commercial antibodies. Tenascin and aggrecan were increased in early OA and slightly decreased in late OA. Complement factor D was decreased in both early and late OA. Thus, western blotting corroborated the results of mass spectrometry analysis (Additional file 1: Table S1).

### Uric acid analyses

Research by Denoble and co-workers recently found uric acid levels to be highly correlated with synovial fluid IL-18 and IL-1β, which paralleled an increase in OA joint pathology [20]. Although other crystals (that is, calcium pyrophosphate dihydrate (CPPD) and basic calcium phosphate hydroxy-apatite (BCP)) have been shown to be associated with joint pathology, the high positive correlation between uric acid levels and OA joint pathology found in the referenced literature provided an interesting and easily attainable accession to our study. Thus, we determined the concentration of uric acid from shoulder SF from healthy subjects and patients with early and late stages of OA. Uric acid analysis of SF failed to show significant differences between the healthy subjects, patients with early OA and patients with late OA. The results obtained for the samples are as follows: 3.08 for pooled healthy, 3.05 ± 0.99 for early (n = 5) and 3.10 ± 1.08 for late (n = 4) samples.

## Discussion

As most studies have focused on knee (weight-bearing joint) OA, less is known about the pathogenesis of shoulder OA (non-weight bearing joint). Thus, we have employed quantitative proteomic techniques to elucidate the pathological pathways of OA in shoulder SF through disease progression. Our results clearly show pathological pathways present in osteoarthritic samples, which exhibit a similar pattern to those present in other osteoarthritic joints, particularly the knee [21]. Comparison of early and late OA samples identified 31 and 38 differentially regulated proteins respectively, many of which have already been identified previously in SF of other osteoarthritic joints [21,22]. This differential profile provides potential insights into the pathophysiology of shoulder OA. Of the differentially regulated proteins, proteins belonging to the complement system, extracellular matrix proteins, serpins and immunoglobulin classes were prominently identified.

Serpins are a superfamily of proteins that inhibit serine proteases. They are known to control processes such as inflammation and coagulation [23,24]. We have identified several serpins in late-OA samples that are downregulated (Figure 4B). Consistent with our result, SERPINA1, the most abundant inhibitor in SF, has been reported to be downregulated in both the early and late OA sets [24]. These results suggest that serine protease activities are unregulated in SF, leading to cartilage destruction in OA [25]. Hence, serpin molecules may be potential therapeutic agents that could help control matrix degradation in OA.

The complement system is known to play a significant role in host defense and inflammation. Overexpression of molecules from the classical complement system is seen in both early OA and late OA (Figure 4). Our results show that complement factor D, which plays a key role in the alternate complement pathway, was downregulated in early OA (Figure 4A). This supports the importance of the classical complement system in shoulder OA which corroborates with a recent study that established a central role of the classical complement system in OA in a mouse model [8].

Early- and late-stage OA is characterized by a loss of proteoglycans. Degeneration of articular cartilage is observed in early OA, and extensive fissuring, fibrillation, clustering of chondrocytes and loss of cartilage is observed in late OA. Historically, a severe loss of extracellular matrix proteins has also been observed in late OA [26]. Our results demonstrated a downregulation of extracellular matrix proteins in both early and late sets (Figure 4). Thus, our results are consistent with earlier findings in SF from horses with induced OA [27].

Increased amounts of aggrecan and cartilage oligomatrix protein (COMP) fragments have been observed in early stages but not in advanced stages [28]. However, a recent study found increased levels of aggrecan and COMP fragments in the SF and serum of knee OA patients [29]. Reduced mRNA levels of aggrecan from osteoarthritic cartilage have been noted earlier [30]. Western blot analysis for aggrecan in early and late OA samples passed through the immunodepletion column gave a single band (Figure 7) while unprocessed samples gave two bands (data not shown). Additional experiments will need to be carried out to investigate the nature of the fragments arising from aggrecan. Increases in the concentration of tenascin (Figure 7) was also observed in early OA sets but was slightly downregulated in late OA sets, which may be the result of the reparative response of the injured tissue, as tenascin is known to be overexpressed in osteoarthritic cartilage according to a previous report [30].

In our study, an increased concentration of apolipoprotein B, the most abundant protein, was observed in early OA sets whereas a less significant change was observed in late OA sets (Figure 4). Increased levels of apolipoprotein B have been observed in earlier knee OA studies [31]. However, one interesting result to point out is that lower levels of paroxonase activity have been reported in OA [32] which corroborates with our results from early OA sets (Figure 4).

Among the proteins that were bound to the high capacity multiple affinity removal spin cartridge, increased alpha 2 macroglobulin, was observed in both early OA and late OA (Additional file 4: Table S4) as seen in a previous study [33]. The protein is known to complex with MMPs [34] which may eventually lead to deterioration of articular cartilage. Our group also observed that immunoglobulin molecules were upregulated in both early and late sets, suggesting underlying joint inflammation.

The present study examined the presence of interleukins in SF of shoulder OA patients (Figure 6). Proinflammatory cytokines, IL-6, IL-8 and IL18, were found to be increased in early and late OA SF. Significant increases in IL6, IL8 and IL18 have been reported in SF of the knee [20,35]. IL-6 is a proinflammatory cytokine that is markedly upregulated during tissue inflammation [36]. In addition, it has been shown to induce proteoglycan degradation and inhibit chondrocyte proliferation [37]. IL-8 is known to promote altered chondrocyte differentiation and calcification in OA [38]. IL18 may induce prostaglandin E2 in SF, thus promoting cartilage damage [39]. Among anti-inflammatory cytokines only IL10 showed a modest increase in SF. Increases in IL-10 in SF of OA patients have also been reported [37], which is known to decrease the production of IL-1β and TNF alpha, and inhibit prostaglandin E2 (PGE2) [39,40].

The results observed with regard to uric acid concentration in healthy, and early- and late-stage OA samples in this study, failed to show significant differences. Our results are in contrast to those found in an earlier paper [20], suggesting that the glenohumeral joint, a non-weight bearing joint, may not accumulate uric acid in a predictive fashion as has been reported in the knee (weight bearing joint). Additional investigation is necessary to understand the biological principles that underlie these differences.

Extracellular matrix proteins like aggrecan and COMP are promising biomarkers in knee OA for early diagnosis [41]. Cartilage degradation proteins, aggrecan, and COMP are downregulated considerably in early OA sets as well as late OA sets. These two proteins may serve as promising diagnostic biomarkers in early diagnosis as well as monitoring therapeutic modalities.

## Conclusion

The presented proteomic study is the first to analyze SF from patients with shoulder OA. Aggrecan and COMP proteins have the potential to be developed as diagnostic biomarkers. A promising lead obtained from our study in understanding shoulder OA, is the expression of protease inhibitors in the late-stage OA sets. Downregulation of serpins may eventually lead to uncontrolled activity of proteases resulting in matrix destruction. Development of specific protease inhibitors as potential therapeutic agents may lessen the destruction of cartilage in shoulder OA. Ultimately, this may help to develop new treatment modalities leading to effective disease management.

## Abbreviations

ABC: Ammonium bicarbonate; CID: Collision-induced dissociation; COMP: Cartilage oligometric matrix protein; EOA: Early osteoarthritis; FDR: False discovery rate; HPLC: High performance liquid chromatography; IE: Iodoethanol; IL: Interleukin; LOA: Late oateoarthritis; LXR/RXR: Liver x receptor/retinoid x receptor; MMP: Matrix metalloproteinase; MR: Magnetic resonance; NCE: Normalized collision energy; OA: Osteoarthritis; 18O: Oxygen-18; PBS: Phosphate-buffered saline; PFOA: Perfluorooctanoic acid; PGE2: Prostaglandin 2; SERPINA1: Serine (or cysteine) protease inhibitor clade A (alpha antitrypsin), member 1; SF: synovial fluid; TEP: Triethyl phosphine; TFA: Trifluoroacetic acid; TNF: Tumor necrosis factor.

## Competing interests

The authors declare that they have no competing interests.

## Authors’ contributions

JPW performed sample collection, preprocessing of the samples for mass spectrometry analysis, data analysis as well as validation studies, and also was the primary author of the manuscript. RSS was involved with preprocessing of the samples for mass spectrometry analysis, data analysis/interpretation, validation studies and bioinformatic studies, and helped to edit the manuscript. YSP designed the western blot analysis and helped edit the manuscript. YS aided in sample collection and contributed to manuscript editing. EB designed and conducted the ELISA analysis and contributed to manuscript editing. SM supervised statistical and pathway analysis of the proteome sets and contributed to manuscript editing. RG was involved with the coordination of the study as well as interpretation of the results, and also contributed to manuscript editing. MM helped to conceive the study, and also contributed to study design and interpretation of experimental results, as well as editing the manuscript. RG conceived the study, provided source material for the study and contributed to interpretation of results, and helped with manuscript editing. All authors read and approved the final manuscript.

## Supplementary Material

Additional file 1: Table S1 Information contained in this table is provided under two separate headers. The first header that starts with *Protein details* provides information about the protein. The second header that begins with *Peptide details* provides information about the peptide identified within Mascot. Click here for file

Additional file 2: Table S2 This table lists the proteins quantified in the five early-stage osteoarthritis (OA) sets versus control experiments and the four late-stage OA versus control experiments. Click here for file

Additional file 3: Table S3 This table lists the proteins found across the early- and late-stage osteoarthritis (OA) samples with statistical analyses as well. Click here for file

Additional file 4: Table S4 This table displays the fold changes of proteins from patients with early osteoarthritis (OA), bound to the high capacity multiple affinity removal spin cartridge. Click here for file

Additional file 5: Table S5 This table displays the differentially expressed proteins in early-and late-stage osteoarthritis (OA) samples with fold changes ≥1.5 and ≤ -1.5. Click here for file
